# 

**Published:** 1892-11-19

**Authors:** 


### CONTENTS.

| | PAGE |
|---|---|
| **Faithfulness and Kingship** | 113 |
| **Passing Topics.**—The Last New Thing in Hospitals | 114 |
| **Medical History from the Earliest Times.** By E. T. Withington, M.A., M.B.Oxon. XXXII.—Arabic Medicine. (5) Hospitals and Institutions | 115 |
| **Diet in Disease.** By Mrs. Ernest Hart. VI.—Restoratives: Coffee, Cocoa, Chocolate | 116 |
| **The Fruits of Vivisection.** By Lawson Tait, F.R.C.S., LL.D., &c. | 118 |
| **Annotations.**—A Horses' Hospital—Is Science too Egotistical?—The Value of R.I.B.A. | 119 |
| **Notes and News** | 120 |
| **The Student of Medicine.**—Vacancies. | |
| **The Hospital Clinic—** | |
| Brompton Consumption Hospital.—The Treatment of Pleurisy | 121 |
| Bath Eye Infirmary.—The Treatment of Granular Lids | 121 |
| Paddington Green Children's Hospital.—The Feeding of Infants and Children. II.—In Disease | 122 |
| The Practitioners' Bookshelf.—Pictorial Medicine | 124 |
| New Drugs, Appliances, and Things Medical.—A New Form of Soda-water Machine | 124 |
| **The Institutional Workshop—** | |
| Within the Asylums.—Berrywood Asylum | 125 |
| Hospital Construction.—Sheffield General Infirmary | 126 |
| Hospitals for the Insane.—Attendants in the Olden Times | 127 |
| **Around the Hospitals.—Scraps and Gleanings** | 128 |

### EXTRA SUPPLEMENT.—THE NURSING MIRROR.

| | |
|---|---|
| **En Passant.**—Whitechapel Infirmary—Weighed in the Scale—Miss Marsden's Book—A Doctor's Views on Nursing—Ipswich Nurses' Home—A Matron's Wedding—Glasgow Royal Infirmary—Short Items | xliii |
| **Lectures for Asylum Attendants.** By William Harding, M.B.—Management of Lunatics (*continued*) | xliv |
| **Nurses' Bookshelf.**—Household Nursing | xlv |
| **The R.B.N.A. and the Lord's Committee's Report** | xlv |
| **Appointments** | xlv |
| **Notes from Australia** | xlvi |
| **Everybody's Opinion.**—Health Classes for the People—Asylum Nurses—Where to Live | xlvi |
| **Wants and Workers** | xlvii |
| **For Reading to the Sick.**—"Fragrance" | xlvii |
| **The Muses' Looking Glass.**—"King Lear" at the Lyceum | xlviii |
| **Notes and Queries** | xlviii |

